# Supplementary figures and images for: The chromatin structuring protein HMGA2 influences human subtelomere stability and cancer chemosensitivity
Source: PLoS One. 2019 May 8;14(5):e0215696. doi: 10.1371/journal.pone.0215696 (PMC6505889; doi:10.1371/journal.pone.0215696)

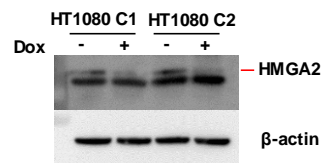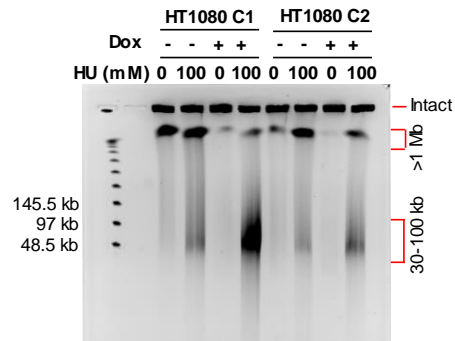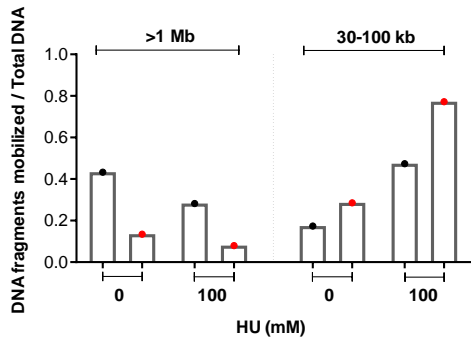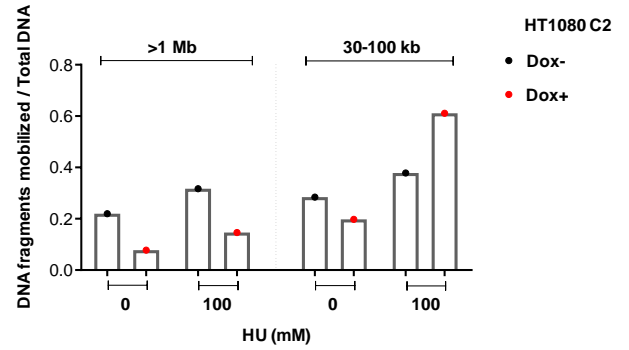

Supplement: S1 Fig — (A) Western blot showing HMGA2 levels in HT1080 C1/C2 clonal cell lines (top panel). HMGA2 expression was down-regulated by doxycycline (Dox)-induced shRNA expression for 96 h in conjunction with HU treatment for the last 24 h [27]. PFGE analysis of DSB formation in response to 24 h incubation with HU (bottom middle panel). Quantification of HU-induced DNA fragments (>1 Mb and 30–100 kb fractions) was done by ImageJ software with each fragment fraction normalized to total DNA loaded (bottom panels). These experiments are independent reproductions of those presented in Yu, Lim (27). (PDF) [file pone.0215696.s001.pdf]

**A**

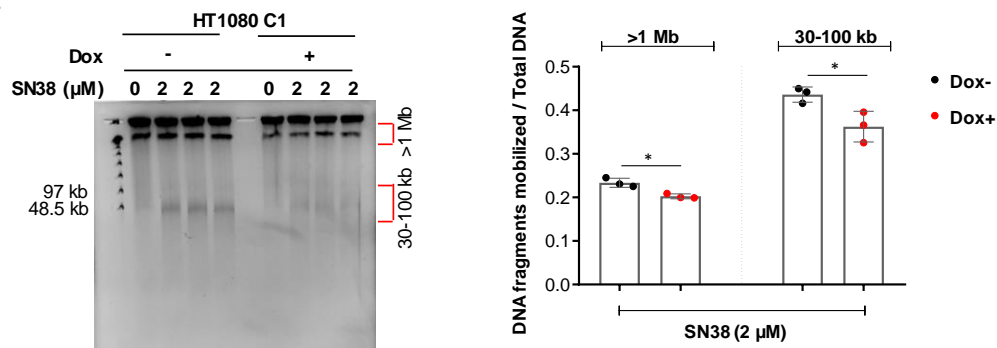

**B**

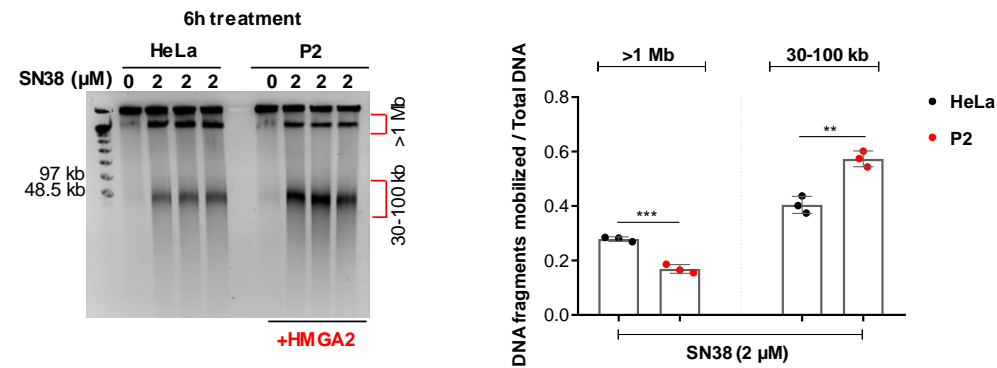

Supplement: S2 Fig — (A) PFGE analysis of DSB formation in HT1080 C1 cell line in response to a 48 h incubation with SN38 (left panel). HMGA2 expression was down-regulated by doxycycline (Dox)-induced shRNA for 96 h. Quantification of SN38-induced DNA fragments (>1 Mb and 30–100 kb fractions) was done by ImageJ software (right panel) with each fragment fraction normalized to total DNA loaded (n = 3 independent experiments). Error bars show SD. Unpaired two-tailed t-tests. * p < 0.05. (B) PFGE analysis of DSB formation in HeLa cells (parental and HMGA2 expressing cell line (P2)) in response to 6 h incubation with SN38 (left panel). Quantification of SN38-induced DNA fragments (>1 Mb and 30–100 kb fractions) was done by ImageJ software (right panel) with each fragment fraction normalized to total DNA loaded (n = 3 independent experiments). Error bars show SD. Unpaired two-tailed t-tests. ** p < 0.01, *** p < 0.001. (PDF) [file pone.0215696.s002.pdf]

**A**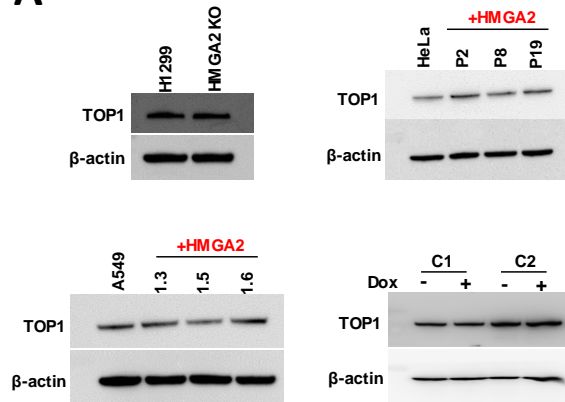**B**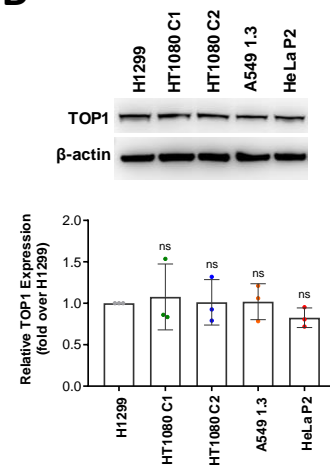**C**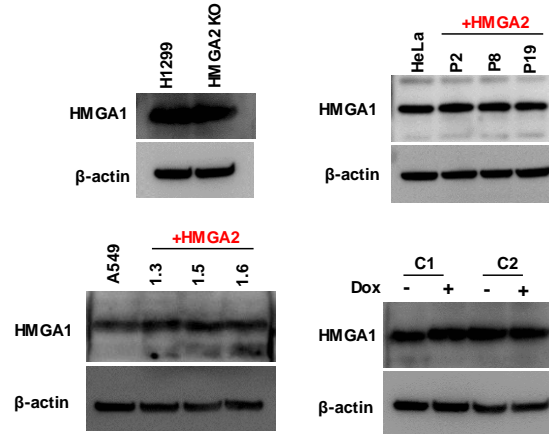**D**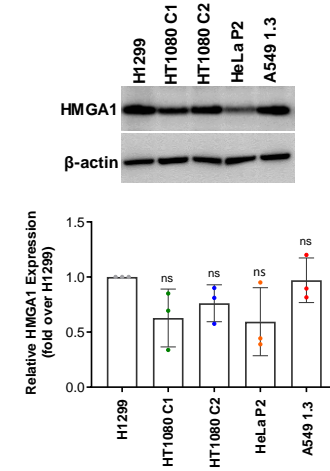**E**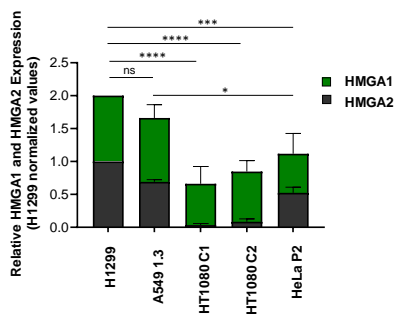**F**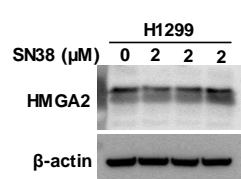**G**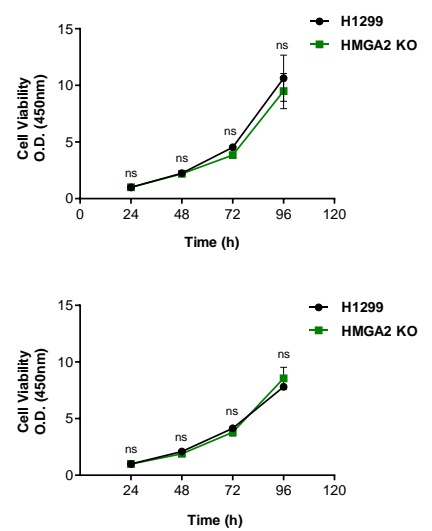

Supplement: S3 Fig — (A) Western blots showing human TOP1 expression across all tested cell lines (H1299 parental and HMGA2 KO cells), A549 cells (parental and three recombinant HMGA2-expressing cell lines), HeLa cells (parental and three recombinant HMGA2-expressing cell lines) and HT1080 C1/C2 (Dox-/+ treated) cells). (B) Representative Western blot comparing human TOP1 expression across various cell lines (top panel). Quantification (bottom panel) of TOP1 expression relative to H1299 cells (set as 1) was done by ImageJ software (n = 3 independent experiments). Error bars show SD. Unpaired two-tailed t-tests. ns not significant. (C) Western blots showing human HMGA1 expression within cell lines, i.e. H1299 (parental and HMGA2 KO cells), A549 cells (parental and three clonal recombinant HMGA2-expressing cell lines), HeLa cells (parental and three clonal recombinant HMGA2-expressing cell lines) and HT1080 C1/C2 (Dox-/+ treated) cells). (D) Representative Western blot comparing HMGA1 expression across various cell lines, as indicated (top panel). Quantification (bottom panel) of HMGA1 expression relative to H1299 cells (set as 1) was done by ImageJ software (n = 3 independent experiments). Error bars show SD. Unpaired two-tailed t-tests. ns not significant. (E) Quantification of combined HMGA1 plus HMGA2 protein expression across various cell lines. Note that the main difference in HMGA expression is contributed by HMGA2. Error bars show SD. Two-way ANOVA followed by Sidak’s multiple comparisons. ns not significant, * p < 0.05, *** p < 0.001, **** p < 0.0001. (F) Western blot showing HMGA2 expression after 2 μM SN38 treatment for 48 h in H1299 cells (3 independent experiments). DMSO treated cells used as experimental control. β-actin was used as a loading control. (G) Cell survival (CCK8) assay in H1299 and HMGA2 KO cells, analyzed for growth differences up to 4 days (n = 2 independent experiments with 3 technical replicates for each time point). Data normalized to the mean of 3 technica [file pone.0215696.s003.pdf]

**A**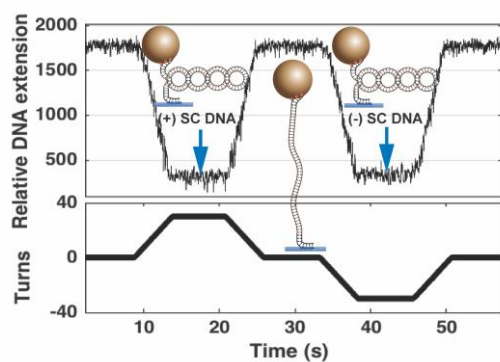**B**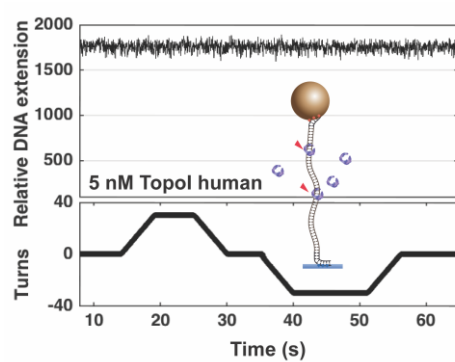**C**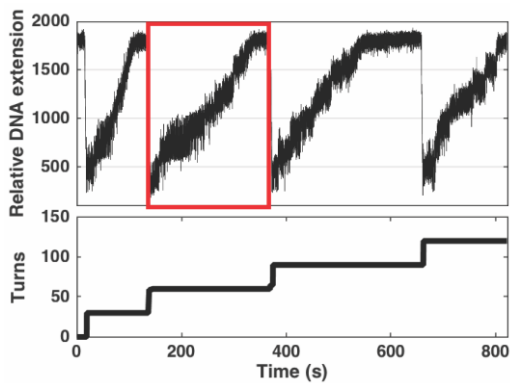**D**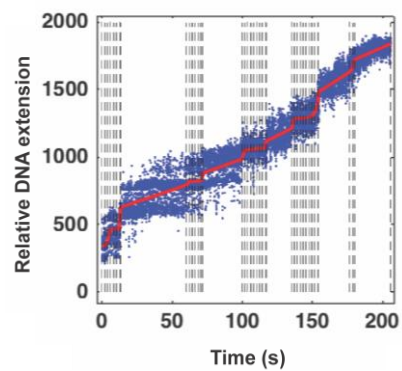**E**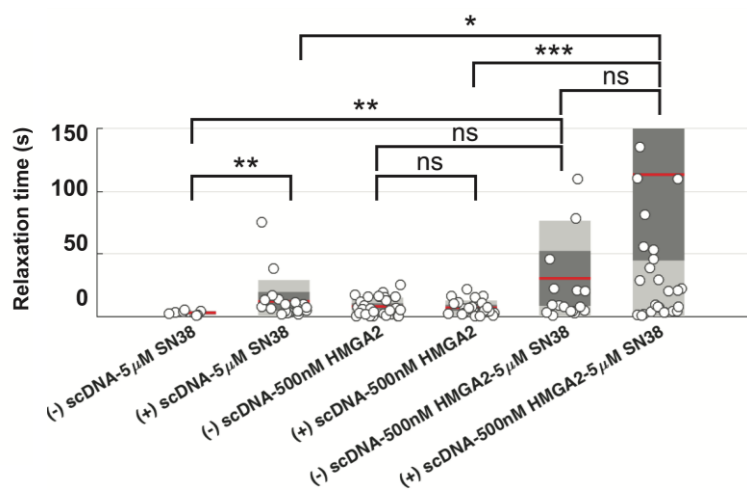

Supplement: S4 Fig — (A) In the absence of SN38, a representative time-trace of extension (top panel) of a torsionally constrained DNA held at 0.3 pN during clockwise and anti-clockwise rotation of the bead (bottom panel). The extension decrease suggests that (+/-) supercoiled DNA is generated by the rotating magnetic beads. Inset: sketch of torsionally constrained DNA in the (+/-) supercoiled and relaxed conformation. (B) In the presence of 5 nM TOP1, DNA extension remains at the level of unconstrained DNA during bead rotations suggesting that topoisomerase I instantaneously and effectively relaxed supercoiled DNA. (C) In the presence of 5 nM TOP1 and 5 μM SN38, slow relaxation of positive supercoiled DNA is observed. When the DNA extension is relaxed to its original length, another 30 turns is applied to the DNA and thus cycles of DNA extension-relaxation events are recorded. (D) The representative relaxation event from (C) highlighted in red box is fitted by piecewise linear regression. (E) Enlarged box plot of relaxation time (grey circle) as shown in Fig 5G to highlight the effects of SN38 or HMGA2 alone on DNA supercoil relaxation by human topoisomerase I. (PDF) [file pone.0215696.s004.pdf]

**A**

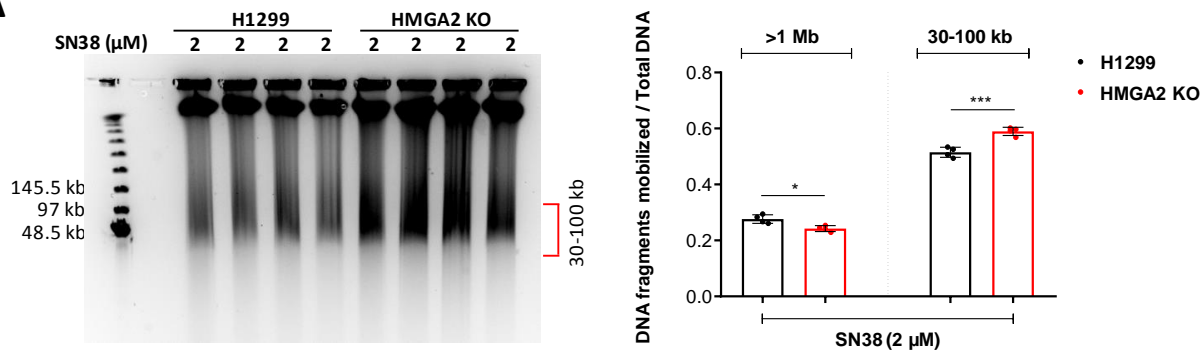

**B**

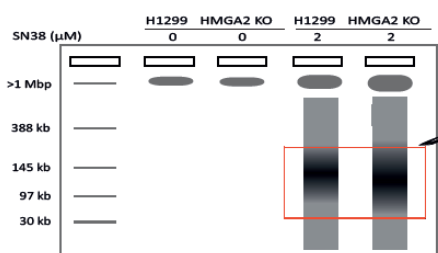

**C**

↓ DNA extraction, sequencing and alignment to GRCh38

GRCh38 chr17

**D**

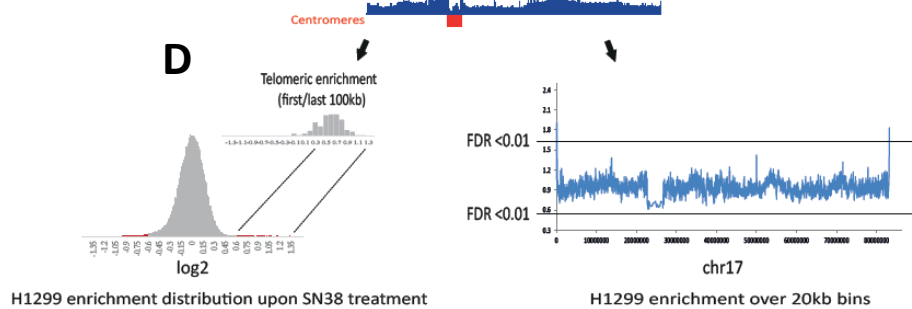

Supplement: S5 Fig — (A) Analysis of DSB formation by PFGE in H1299 cells (parental and HMGA2 KO) in response to 48 h incubation with SN38 (left panel). Quantification of SN38-induced DNA fragments (>1 Mb and 30–100 kb fractions) was done by ImageJ software (right panel) with each fragment fraction normalized to total DNA loaded (n = 4 independent experiments). Error bars show SD. Unpaired two-tailed t-tests. * p < 0.05, *** p < 0.001. The 30–100 kb fragments were gel extracted, combined and sequenced for each cell line. (B-D) Sequencing Workflow. (B) Scheme of representative PFGE image highlighting extracted fragments (red box). (C) Sequencing reads aligned to GRCh38, and coverage of chr17 is shown as an example. (D) Enrichment ratio for each bin was calculated (treated versus untreated samples), and the data for H1299 is shown (left panel). Values plotted for chr17 with FDR <0.01 as cut-off for enrichment/depletion (right panel). (PDF) [file pone.0215696.s005.pdf]

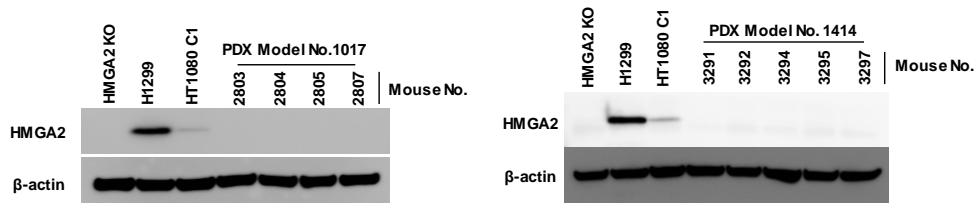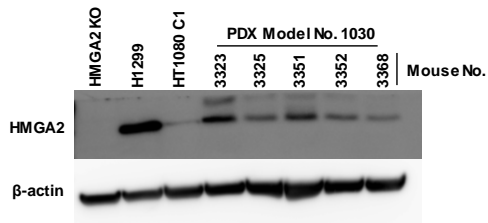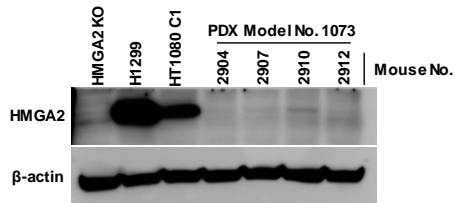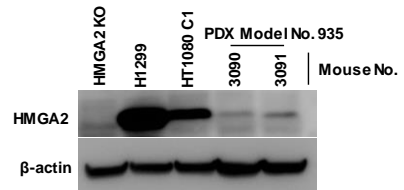

Supplement: S6 Fig — Western blots using the anti-HMGA2 antibody (Ab41878), showing HMGA2 expression of individual PDX tumor models (horizontal numbers) in the corresponding propagating mice (vertical numbers) in comparison with HMGA2 expressing cell lines, as indicated. β-actin was used as a loading control. Note that there is variation in HMGA2 expression in different mice used for propagation of model 1030. (PDF) [file pone.0215696.s006.pdf]
